# Thoracic endovascular aortic repair with collateral vessels from the femoral artery to Adamkiewicz’s artery

**DOI:** 10.1093/icvts/ivad006

**Published:** 2023-01-13

**Authors:** Kazuki Noda, Yoshimasa Seike, Tatsuya Nishii, Hitoshi Matsuda

**Affiliations:** Department of Cardiovascular Surgery, National Cerebral and Cardiovascular Center, Suita, Japan; Department of Cardiovascular Surgery, National Cerebral and Cardiovascular Center, Suita, Japan; Department of Radiology, National Cerebral and Cardiovascular Center, Suita, Japan; Department of Cardiovascular Surgery, National Cerebral and Cardiovascular Center, Suita, Japan

**Keywords:** Adamkiewicz’s artery, Collateral pathway, Abdominal approach

## Abstract

Identification of the Adamkiewicz’s artery (AKA) prior to the operation is one of the spinal cord ischaemia preventive measures. A 75-year-old man presented with the rapid expansion of thoracic aortic aneurysm. Collateral vessels from the right common femoral artery to the AKA were observed on preoperative computed tomography angiography. The stent graft was successfully deployed through the contralateral side via a pararectal laparotomy to avoid collateral vessel injury supplying the AKA. This case highlights the significance of preoperative identification of collateral vessels to the AKA.

## INTRODUCTION

The Adamkiewicz’s artery (AKA) is the dominant spinal cord feeder, and the importance of AKA identification has been reported [1]. Here, we report a rare case of thoracic endovascular aortic repair (TEVAR) with collateral vessels from the common femoral artery (CFA) to the AKA.

## CASE REPORT

**Figure 1:**
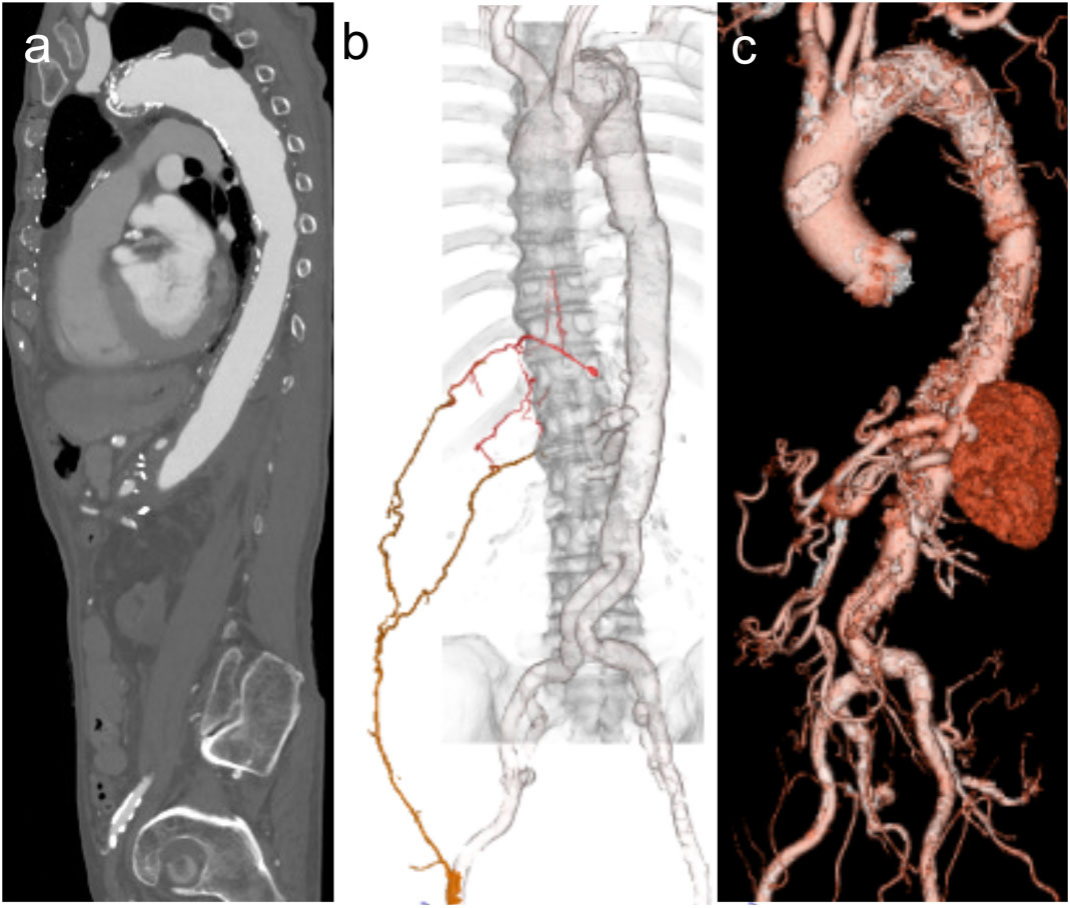
(**a**) Computed tomography angiography revealed an aneurysm in the distal aortic arch within the sagittal plane. (**b**) Collateral vessels to the Adamkiewicz’s artery via the right 11th intercostal artery from the right common femoral artery. (**c**) Postoperative three-dimensional computed tomography revealed no contrast effects into the aneurysmal sac and no access route injury.

A 75-year-old man with end-stage renal disease requiring haemodialysis presented with a distal aortic arch saccular aneurysm which was 52 mm in diameter (Fig. 1a). His past medical history included abdominal aortic replacement 11 years ago and a thoraco-abdominal aortic aneurysm repair from 6th thoracic vertebral level to the previous implanted bifurcated graft with left 11th intercostal artery reconstruction 2 years ago, which was diagnosed as a feeder to AKA. Computed tomography angiography revealed that the left 11th intercostal artery side branch was occluded and the AKA was visualized from the right CFA via the right iliac circumflex artery and right 11th intercostal artery (Fig. 1b). TEVAR with zone 3 landing was suspected as the saccular aneurysm showed a rapid 5-mm expansion in 6 months.

**Figure 2:**
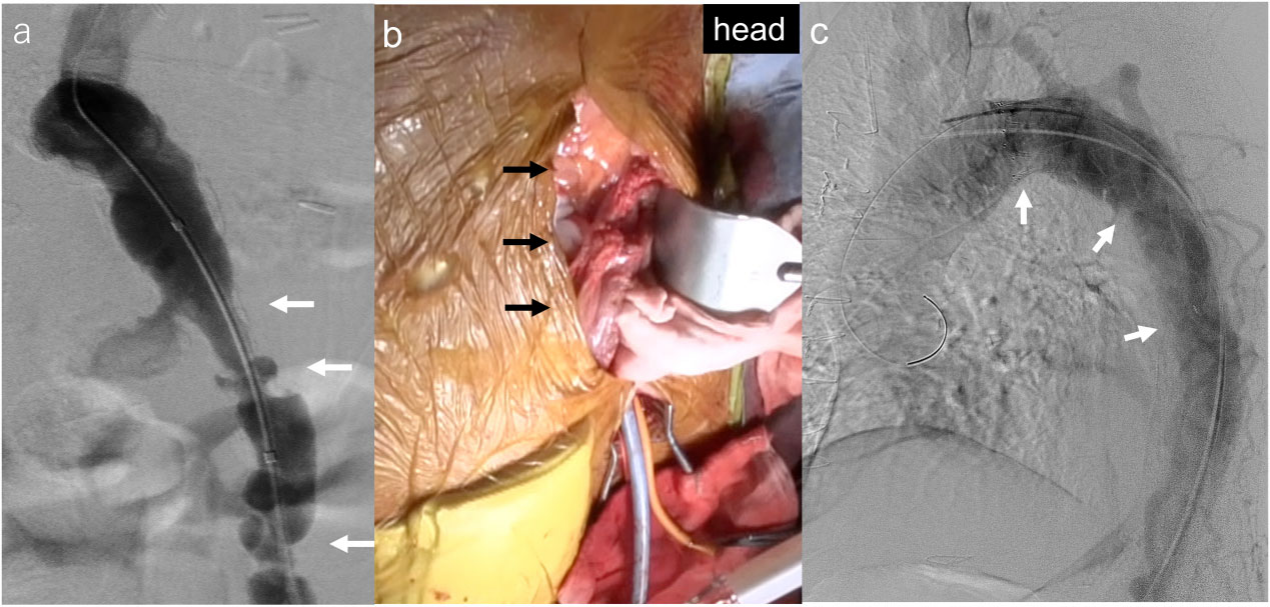
(**a**) Angiography revealed multiple arterial stenoses (white arrows) of the left external iliac artery. (**b**) The approach of thoracic endovascular aortic repair was converted from femoral access to the left leg of the abdominal bifurcated graft through a left pararectal laparotomy (black arrows). (**c**) The stent graft was deployed (white arrows) at the 4th–9th thoracic vertebral level and no endoleak was residual.

TEVAR was performed under general anaesthesia. Motor-evoked potential (MEP) was monitored for early spinal cord ischaemia (SCI) detection. Left CFA was determined as the access site to avoid SCI due to right CFA injury and/or embolism caused due to catheter manoeuvres inside the right iliac and femoral artery. Arterial stenoses with circumferential calcification were detected at the left external iliac artery (Fig. 2a); transluminal balloon angioplasty was performed. A 20-Fr DrySeal Flex Sheath (W.L. Gore & Associates, Flagstaff, AZ, USA) was unable to pass the stenosis to confirm low-profile stent graft passage. Eventually, the left leg of the abdominal bifurcated graft was exposed and a 24-Fr DrySeal Flex Sheath was directly inserted via a pararectal laparotomy (Fig. 2b). The RelayPro thoracic stent graft system (21 Fr, 40 mm × 36 mm × 150 mm; Terumo Aortic, Bolton Medical Inc, USA) was deployed at the 4th–9th thoracic vertebral level (Fig. 2c). Aortography revealed no endoleak and MEP did not decline throughout the procedure.

The postoperative course was uneventful and no neurological deficits developed.

## DISCUSSION

SCI is a devastating TEVAR complication, with a reported incidence range of 3–10% [2]. A multi-disciplinary approach to prevent SCI was reported [1] since preoperative computed tomography angiography was introduced as a reliable method to visualize AKA [3]. As a measure to prevent SCI, prophylactic cerebrospinal fluid drainage (CSFD) has been considered prior to thoraco-abdominal replacement. However, our current institutional review revealed that prophylactic CSFD is less significant when the risk factor for SCI is not significant in simple TEVAR, and prophylactic CSFD was not indicated in the present case [4]. Other routine measure, augmentation of systemic blood pressure, was achieved by the aggressive use of catecholamines to raise mean blood pressure above 80–90 mmHg after deploying the endograft and to increase blood supply to the spinal cord through collateral vessels, especially in patients at the high risk of SCI or with abnormal intraoperative MEPs.

Preoperative AKA identification is more important in patients with previous thoracic aortic surgery due to the risk of development of collateral vessels [5]. This patient presented with the CFA as a collateral AKA source, which is extremely rare. Preoperative confirmation would allow SCI prevention caused by vessel injury.

## CONCLUSION

Preoperative identification of rare collateral vessels to the AKA from the right CFA contributed for the optimal access site determination.

**Conflict of interest:** none declared.

## Reviewer information

Interdisciplinary CardioVascular and Thoracic Surgery thanks Tomislav Kopjar, Giampiero Esposito and the other, anonymous reviewer(s) for their contribution to the peer review process of this article.
